# Alumni Association, University of Buffalo—Department of Medicine

**Published:** 1904-06

**Authors:** Thomas H. McKee

**Affiliations:** ’98 Secretary


### Alumni Association, University of Buffalo — Department of Medicine.

*Twenty-Ninth Annual Meeting, May 3, 1904.*

Reported by THOMAS H. McKEE, '98, Secretary.

Morning Session.

The morning session was held in the college building and was devoted to registration and class reunions, opening at 9.30 a. m. The following mentioned classes held meetings: '54 in the faculty room, John G. Collver, president, Waterford, Ont.; '64 in the library, Clayton L. Hill, president, 321 Franklin street, Buffalo,

N. Y.; '74 in the museum, Edward N. Brush, president, Sheppard and Enoch Pratt Hospital, Towson, P. O., Md.; '84 in the amphitheater, Charles S. Payne, president, Liberty, N. Y.; '94 in alumni hall, Myron E. Fisher, president, Delevan, N. Y. These reunions adjourned at 11 a. m. to attend the commencement exercises, held at the Teck Theater.

### Afternoon Session.

The regular session was called to order in Alumni Hall by the president, A. W. Henchell, of Rochester, at 2.30 o'clock, May 3, 1904.

After hearing the minutes of the previous session, and receiving reports of various committees, the president appointed the nominating committee as follows: William Warren Potter, '59, Buffalo; Edward N. Brush, '74, Baltimore, Md.; Fridolin Thoma, '87, Buffalo. The committee reported later as follows:

*To the Alumni Association, University of Buffalo:*

Your committee of nomination begs to report as follows: for president, De Lancey Rochester, '84, Buffalo; for first vice-president, Fridolin Thoma, '87, Buffalo; for second vice-president, Henry T. Benham, '90, Honeoye Falls, N. Y.; for third vice-president, Jane W. Carroll, '91, Buffalo; for fourth vice-president, G. A. Himmelsbach, '91, Buffalo; for fifth vice-president, C. J. Reynolds, '90, Buffalo; for secretary, Thomas H. McKee, '98, Buffalo; for treasurer, H. K. DeGroat, '97, Buffalo.

To fill vacancies on the board of trustees: Robert P. Bush, '74, Horseheads, N. Y., vice William Warren Potter, resigned; A. W. Henchell, '89, Rochester, vice D. A. Currie, term expired.

For members of the executive committee: Albert T. Lytle, '93, chairman, Buffalo; Grover W. Wende, '89, Buffalo; Franklin W. Barrows, '93, Buffalo.

In submitting the foregoing report your committee desires to supplement it with the statement that the address of the president was listened to with interest, particularly that portion referring to the apparent lack of interest in the annual meetings. Your committee feels that the condition referred to is more apparent than real, and that it does not indicate a waning affection on the part of the graduates for their Alma Mater.

The committee feels all that is needed is something to awaken an increased interest in the annual sessions, and that the suggestions of the president are of decided value in this direction. It, therefore, recommends that the executive committee confer at an early date with the faculty with a view of making a session of at least three days duration for the next reunion, and that the suggestions of the president as to clinics and other matters be given the weight which their importance seems to justify.

Respectfully submitted,

William Warren Potter,
Edward N. Brush,
Fridolin Thoma.

On motion of Dr. Thoma, seconded by Dr. Brush, the report was received, adopted, and the nominations were confirmed.

The auditing committee, Franklin W. Barrows, Elmer G. Starr, and Jane W. Carroll, reported as follows:

May 3, 1904.

Your committee has examined the treasurer's report and the vouchers accompanying it and finds all accounts correct. We also heartily endorse the recommendation of the treasurer, that some action be taken that may render it unnecessary to carry the accounts of delinquent members from year to year.

Franklin W. Barrows, '93.
Elmer G. Starr, '84,
Jane W. Carroll, '91.

The recommendation of the committee was discussed by Drs. Stockton, Currie, Lytle, and Rochester. On motion of Dr. Currie, seconded by Dr. B. G. Long, the treasurer was instructed to make the best settlement possible with members in arrears for dues.

Moved by Dr. Lytle, seconded by Dr. Stockton, that the executive committee be instructed to make arrangements for a three days' meeting in 1905. Carried. Dr. Currie suggested that the session begin one day before commencement.

The meeting was then presented with the regular program for the afternoon.

1. President's address, A. W. Henchell, '89. Discussion.
2. Tubercular pericarditis, Charles G. Stockton, '78, professor of medicine, medical department. Discussion.
3. The suprarenal gland, Frederick A. Busch, '97, professor of physiology, medical department. Discussion.
4. Excreted nitrogen, Herbert M. Hill, professor of chemistry, medical department. Discussion.

The paper entitled Recent studies on the tubercle bacillus and its relation to the tuberculosis problem, by Herbert U. Williams, '89, professor of pathology, medical department, was on motion of the author, owing to the lateness of the hour, indefinitely postponed. At the evening session the annual banquet was served at the Hotel Iroquois at 6.30 o'clock.

